# Predictive Value of Updating Framingham Risk Scores with Novel Risk Markers in the U.S. General Population

**DOI:** 10.1371/journal.pone.0088312

**Published:** 2014-02-18

**Authors:** Bart S. Ferket, Bob J. H. van Kempen, M. G. Myriam Hunink, Isha Agarwal, Maryam Kavousi, Oscar H. Franco, Ewout W. Steyerberg, Wendy Max, Kirsten E. Fleischmann

**Affiliations:** 1 Department of Epidemiology, Erasmus MC, Rotterdam, the Netherlands; 2 Department of Radiology, Erasmus MC, Rotterdam, the Netherlands; 3 Department of Health Policy and Management, Harvard School of Public Health, Boston, Massachusetts, United States of America; 4 Department of Epidemiology, Harvard School of Public Health, Boston, Massachusetts, United States of America; 5 Department of Public Health, Erasmus MC, Rotterdam, the Netherlands; 6 Institute for Health & Aging and Department of Social and Behavioral Sciences, University of California San Francisco, San Francisco, California, United States of America; 7 Division of Cardiology, University of California San Francisco, San Francisco, California, United States of America; Scientific Directorate, Bambino Hospital, Italy

## Abstract

**Background:**

According to population-based cohort studies CT coronary calcium score (CTCS), carotid intima-media thickness (cIMT), high-sensitivity C- reactive protein (CRP), and ankle-brachial index (ABI) are promising novel risk markers for improving cardiovascular risk assessment. Their impact in the U.S. general population is however uncertain. Our aim was to estimate the predictive value of four novel cardiovascular risk markers for the U.S. general population.

**Methods and Findings:**

Risk profiles, CRP and ABI data of 3,736 asymptomatic subjects aged 40 or older from the National Health and Nutrition Examination Survey (NHANES) 2003–2004 exam were used along with predicted CTCS and cIMT values. For each subject, we calculated 10-year cardiovascular risks with and without each risk marker. Event rates adjusted for competing risks were obtained by microsimulation. We assessed the impact of updated 10-year risk scores by reclassification and C-statistics. In the study population (mean age 56±11 years, 48% male), 70% (80%) were at low (<10%), 19% (14%) at intermediate (≥10–<20%), and 11% (6%) at high (≥20%) 10-year CVD (CHD) risk. Net reclassification improvement was highest after updating 10-year CVD risk with CTCS: 0.10 (95%CI 0.02–0.19). The C-statistic for 10-year CVD risk increased from 0.82 by 0.02 (95%CI 0.01–0.03) with CTCS. Reclassification occurred most often in those at intermediate risk: with CTCS, 36% (38%) moved to low and 22% (30%) to high CVD (CHD) risk. Improvements with other novel risk markers were limited.

**Conclusions:**

Only CTCS appeared to have significant incremental predictive value in the U.S. general population, especially in those at intermediate risk. In future research, cost-effectiveness analyses should be considered for evaluating novel cardiovascular risk assessment strategies.

## Introduction

Cardiovascular disease (CVD) remains the leading cause of death in the U.S. population [1]. Current guidelines recommend aggressive risk modifying treatment regimens in apparently healthy individuals deemed to be at high cardiovascular risk [2]. These individuals can be identified using risk scores based on traditional risk factors as defined by the Framingham Heart Study [3], [4]. However, the accuracy of Framingham risk scores (FRS) for predicting CVD outcomes can be improved by adding novel risk markers, including imaging techniques and biomarkers.

Recently, the U.S. Preventive Services Task Force and the American College of Cardiology Foundation/American Heart Association Task Force published recommendations on which novel risk markers to use for cardiovascular risk assessment [5], [6]. Four novel risk markers that are expected to have added predictive value beyond the FRS are: the CT coronary artery calcium score (CTCS), high-sensitivity C-reactive protein (CRP), the ankle-brachial index (ABI) and measurement of carotid intima-media thickness (cIMT). Most importantly, studies should have demonstrated that risk assessment including these novel markers should correctly reclassify individuals into clinically relevant risk categories. These risk categories are defined by 10-year risk: e.g. <10% (low risk), 10–19% (intermediate risk) and ≥20% (high risk).

Due to heterogeneous results [7]–[9] and selection of study populations it remains difficult to generalize from published cohort studies that adding these novel markers to the FRS would indeed lead to improved classification in the U.S. population as a whole [10]. In order to synthesize the existing evidence quantitatively, computer simulation modeling with data input from meta-analyses combined with study data representative of the entire population overcomes a number of these limitations [11].

In this study, we aimed to update traditional 10-year FRSs by the published independent associations of CTCS, cIMT, CRP, and ABI with cardiovascular events. Our final purpose was to assess to what extent the predictive value of traditional risk assessment would be improved by these four novel markers in asymptomatic participants of the National Health and Nutrition Examination Survey (NHANES), a cross-sectional study designed to be a representative sample of the U.S. general population.

## Methods

### Systematic Review of the Novel Risk Markers' Predictive Effects

We adopted two recent individual-level meta-analyses for the association of a one unit SD (1.11) log mg/L increase of CRP, and the association of a 0.1 mm increase in mean cIMT with coronary heart disease (CHD) and stroke event rates [12], [13]. Both were adjusted for traditional risk factors. For CTCS and ABI, we updated the 2009 systematic review by the USPSTF [14] through April 19, 2013 (for detailed search syntaxes and study inclusion criteria see the Text S1). Two reviewers independently included potentially eligible articles based on title and abstract. Only studies that recruited subjects from the general population, and which excluded or adjusted for prior CHD and stroke were included. Articles were included if both reviewers agreed that the study design was a cohort, nested case-control, or case-cohort study. Also, systematic reviews that included these study types were considered. Relative risk estimates had to be calculated for CHD and/or stroke, with CHD defined as myocardial infarction or coronary death. We excluded studies that analyzed the novel risk marker with adjustment for less than 5 of the 8 Framingham risk factors: age, sex, smoking, systolic blood pressure, antihypertensive drug therapy, total cholesterol, high density (HDL) cholesterol and diabetes mellitus. One reviewer extracted the reported relative risks and 95% CI limits of an increase in 1 unit log (CTCS+1) for CTCS, and of an ABI≤0.90 vs. >0.90. If relative risks were reported using other units, these were converted in order to match the aforementioned units (see the Text S1 for details). Data extraction was checked by a second reviewer. We used the R ‘meta.summaries’ function of the ‘rmeta’ package to compute summary estimates and 95% CIs by random-effects modeling. Heterogeneity was assessed statistically with the Woolf's test where values <0.05 indicate significant heterogeneity.

### Study Population

We selected data on 3,736 individuals aged 40 or older without a history of myocardial infarction or stroke at baseline from the 2003–2004 NHANES exam, taking into account the sampling weights. We used the following datasets: NHANES 2003–2004 Demographics Data, NHANES 2003–2004 Examination Data, NHANES 2003–2004 Laboratory Data, and NHANES 2003–2004 Questionnaire Data, see http://wwwn.cdc.gov/nchs/nhanes/search/nhanes03_04.aspx. We included the following variables: age at the exam visit, sex, current smoking, systolic blood pressure, total cholesterol, HDL cholesterol, fasting plasma glucose level, anti-diabetic treatment, antihypertensive treatment, ankle-brachial index, and high-sensitivity C-reactive protein. Because values for CTCS and cIMT were not measured in the NHANES study, we merged the NHANES dataset with a subset of the Rotterdam Study Cohort. The Rotterdam Study is a population-based cohort study of individuals aged 55 years and older living in Rotterdam, the Netherlands [15]. Baseline examinations were performed between 1990 and 1993 (Rotterdam Study-I). Traditional FRS risk factors, CTCS, cIMT, hs-CRP, ABI, and information on cardioprotective drugs were simultaneously measured during the third examination round (1997 to 1999) in a subset (n = 1,915) of the Rotterdam Study-I cohort. Details on how these novel risk markers and the other variables were measured are published elsewhere [16], [17]. We imputed the missing CTCS and cIMT values of NHANES subjects within the merged dataset. For the imputation, we used a flexible additive imputation model including all other variables. After the imputation, only NHANES individuals were selected for the analysis (see Table 1 for baseline characteristics, and Text S1 for details on the dataset preparation.

**Table 1:** Baseline characteristics of 3,736 NHANES and 1,915 Rotterdam Study individuals.

Variable	NHANES Median [IQR]	RS Median [IQR]
Age	53 [46–63]	70 [66–75]
Sex (%male)	48%	45%
Current Smoking	23%	16%
Systolic blood pressure (mm Hg)	125 [115–139]	140.0 [124–155]
HRX	27%	28%
Total cholesterol (mg/dl)	209 [183–235]	225 [203–250]
HDL cholesterol (mg/dl)	51 [42–63]	51 [43–62]
Glucose (mg/dl)	97 [90–106]	99 [94–110]
Anti diabetic medication	8%	6.2%
**CTCS** *		
0	37%	10%
1–100	36%	41%
101–400	14%	23%
400–1000	8%	15%
≥1000	5%	11%
Natural logarithm of (CTCS+1)	2.6 [0–4.8]	4.81 [2.6–6.3]
cIMT (mm)*	0.78 [0.69–0.93]	0.86 [0.76–0.95]
CRP (mg/L)	2.1 [0.9–4.6]	2.4 [1.2–4.4]
ABI≤0.9	5.0%	15.6%

Abbreviations: CTCS, CT coronary artery calcium score; HDL, high-density lipoprotein; HRX, antihypertensive drug treatment; NHANES, National Health and Nutrition Examination Survey; RS, Rotterdam Study.

SI conversion factors: To convert CRP to nanomoles per liter, multiply by 9.524; HDL and total cholesterol to millimoles per liter, multiply by 0.0259.

* Imputed by multivariable algorithms.

### Updating Framingham Risk Scores

For both the 10-year cardiovascular risk assessment and simulation of event rates, we used the 30-year FRS as basis for our models [18]. It uses the 8 aforementioned traditional risk factors to calculate 30-year cumulative incidences for both CVD and non-CVD deaths, while taking into account competing risks. CVD is defined as myocardial infarction, coronary death and stroke, non-CVD death is defined as mortality due to all causes other than CVD. In order to calculate CHD and stroke risks separately, we applied a sex-specific ratio of the reported CHD to stroke events to the baseline CVD survival function. For men, the CHD: stroke event ratio was 348/104 and for women it was 133/86. We assumed that the reported regression coefficients of the traditional risk factors were similar for CHD and stroke. To resemble currently recommended risk assessment, we calculated 10-year CVD and CHD risks without adjustment for competing risk. We used the baseline CHD and CVD survival probability at year 10 and subsequently updated the traditional FRS with one novel risk marker at a time. We recalibrated the baseline survival probability by assuming no change in the average survival probability. For both 10-year CVD and CHD, the different models (FRS only, FRS+CTCS, FRS+IMT, FRS+CRP, and FRS+ABI) were used to classify the 3,673 NHANES subjects into to the following risk categories: <10%, ≥10–<20%, ≥20%. In addition, we also classified into <6%, ≥6–<20%, ≥20%: categories [19].

### Cardiovascular outcomes

To simulate cardiovascular event rates, we constructed a state-transition model using TreeAge software (2009 version, TreeAge Software, Inc., Williamstown, MA, USA), consisting of three health states: ‘Well’, ‘Post-CVD’ and ‘Dead’ (see Table S1 for input parameters). A one-year cycle length was used. One-year transition-probabilities were based on the 30-year FRS updated with all four novel risk markers together, assuming independency of predictive effects. We recalibrated the baseline survival function through 30 years of follow-up, while ascertaining that the average 30-year cumulative incidences for CVD and non-CVD death calculated by the state-transition model were equal to the average risks calculated by the original 30-year FRS for the NHANES study sample (see the eMethods for details).

### Predictive Value of the Four Updated Risk Scores

Reclassification tables were created by cross-tabulating NHANES individuals using the three risk categories of the traditional and each updated FRS. Occurrences of events within these individuals were modeled through a state-transition model using Monte Carlo microsimulation. We calculated risks in subjects reclassified upwards and downwards for both cases and non-cases and calculated the net reclassification improvement (NRI) applicable to survival and competing risk data [20]. For the intermediate risk category, we calculated a bias-corrected NRI [21]. In addition, long-term 30-year risks were reported in the reclassification tables to evaluate whether those who are reclassified have a long-term risk that is in agreement with the reclassification. To further assess the models' discriminative performance, we calculated the Harrell's C-statistic [22] using simulated 10-year time-to-event data. To take into account the uncertainty of the hazard ratios of the novel risk markers, 95% CIs were calculated by randomly sampling from lognormal distributions defined by the summary estimates and standard errors taken from the meta-analyses.

### Ethics Statement

For the 2003–2004 NHANES, Institutional Review Board (IRB) approval and documented consent was obtained from all participants (Protocol #98-12). The Rotterdam Study has been approved by the institutional review board (Medical Ethics Committee) of the Erasmus Medical Center and by the review board of The Netherlands Ministry of Health, Welfare, and Sports. The approval has been renewed every 5 years.

## Results

### Systematic Review of the Novel Risk Markers' Predictive Effects

**Figure 1:**
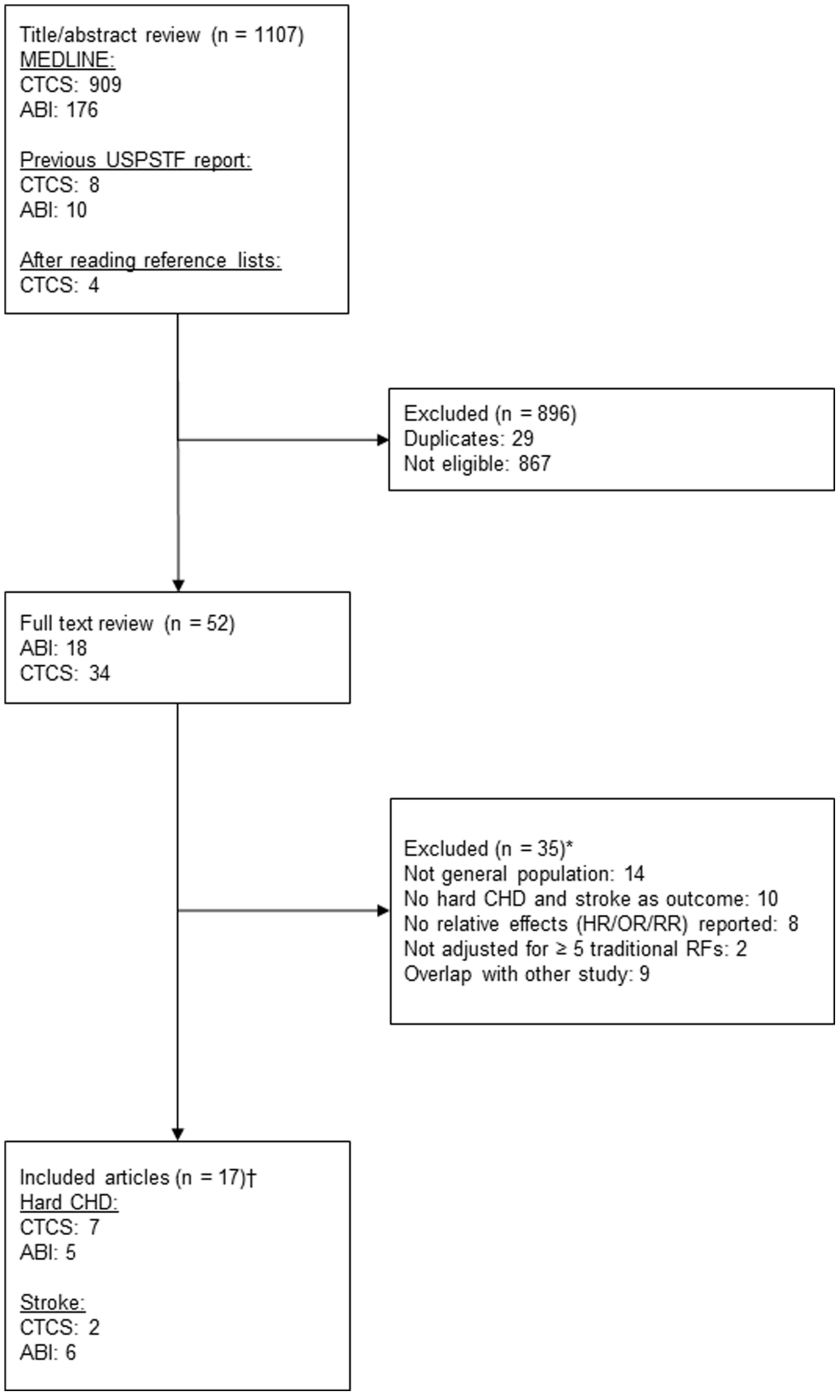
Literature search and selection. Numbers of articles of each step of the review process are indicated. *Group total exceed the reported number for the excluded articles because several reasons for exclusion were allowed. †Group total exceed the number for the included articles, because one article may include estimates for both CHD and stroke. Abbreviations: ABI, ankle-brachial index; CHD, coronary heart disease; CTCS, computed tomography calcium scoring; HR, hazard ratio; OR, odds ratio; RR, risk ratio; USPSTF, United States Preventive Services Task Force.

From the USPSTF report [14], eight studies on CTCS and ten studies on ABI were included in our review. For ABI, we did not use the reported estimates on CHD and stroke, because these were based on a comparison between an ABI≤0.9 and 1.11–1.40 instead of ≤0.9 vs. >0.9 [23]. Combined with the citations found through our additional search, in total 1,107 citations were included in our systematic review. Seventeen articles were used for the data extraction; for reasons of exclusions see Figure 1. In 11 of the articles the effect of the novel risk marker was adjusted for seven or more Framingham risk factors (for the study details see Table S2).

For the association between CTCS and CHD, we performed a meta-analysis on a total of 30,945 individuals and 548 events. Only two studies were found on the association of CTCS with stroke, comprising 7,118 subjects and 117 stroke events. For the ABI meta-analyses, 21,122 subjects with 1,206 CHD events and 36,941 subjects with 987 stroke events were used. One study on the association between ABI and CHD also counted angina as a CHD event [24]. As the authors explicitly stated that the analysis limited to hard CHD events (i.e., excluding angina) showed similar results, we included this study in the analysis. Summary estimates from the meta-analyses are given in Table 2. We found no statistical evidence for heterogeneity between studies. The forest plots are included in the Figures S1, S2, S3, S4.

**Table 2:** Hazard Ratios and confidence intervals from the meta-analyses.

Novel risk marker	HR [95% CI] for CHD	HR [95% CI] for Stroke	Source
Log(CTCS+1)	1.35 [1.28–1.43]	0.97 [0.84–1.12]	This manuscript
0.1 mm IMT	1.08 [1.05–1.10]	1.12 [1.10–1.15]	Den Ruijter et al. [13]
Log(CRP)/SD* (mg/L)	1.22 [1.17–1.27]	1.16 [1.10–1.27]	Emerging Risk Factors Collaboration [12]
ABI≤0.9	1.47 [1.18–1.84]	1.26 [1.05–1.50]	This manuscript

* Pooled SD = 1.11 mg/L.

### Predictive Value of the Four Updated Risk Scores

Most NHANES subjects were at low (<10%) 10-year CVD and CHD risk: respectively 2,641 (71%) and 2,999 (80%). The number of NHANES subjects with intermediate (≥10–<20%) risk was limited: 697 (19%) for CVD and 525 (14%) for CHD as the outcome (see Table 3, and Tables S3 and S4). These numbers approximately doubled with using the alternative threshold values ≥6–<20% to 1385 (37%) for CVD and 1075 (29%) for CHD.

**Table 3:** Ten-year cardiovascular disease (CVD) risk reclassification by CTCS.

	FRS+CTCS			Overall
FRS	<10%	≥10–<20%	≥20%	
**<10%**				
N	2520.53	116.06	4.41	2641
% Events [ 95% CI ]				
10 yr CVD	2.5 [1.9–3.2]	11.2 [6.1–16.6]	19.7 [0–85.8]	2.9 [2.3–3.5]
30 yr CVD	14.8 [13.0–16.2]	49.2 [39.8–57.3]	68.6 [0–100]	16.4 [15.0–18.0]
**≥10–<20%**				
N	240.28	309.36	147.36	697
% Events [ 95% CI ]				
10 yr CVD	6.7 [3.5–9.6]	12.9 [9.0–16.4]	24.8 [17.7–31.4]	13.3 [10.8–16.0]
30 yr CVD	32.5 [27.0–38.9]	50.5 [45.5–55.9]	69.3 [60.9–77.6]	48.3 [43.6–51.9]
**≥20%**				
N	6.62	80.72	310.66	398
% Events [ 95% CI ]				
10 yr CVD	9.7 [0–42.9]	13.9 [7.8–21.3]	40.3 [33.0–47.9]	34.4 [28.8–40.6]
30 yr CVD	33.7 [0–75.0]	48.4 [39.9–58.2]	74.1 [68.3–78.9]	68.2 [63.2–72]
**Overall**				
N	2767.43	506.14	462.43	3736
% Events [ 95% CI ]				
10 yr CVD	2.8 [2.3–3.5]	12.7 [9.6–15.4]	35.2 [30.0–40.0]	8.2 [7.3–40.0]
30 yr CVD	16.4 [14.8–17.8]	49.8 [45.0–54.3]	72.5 [67.6–76.7]	27.9 [26.3–76.7]

Classification on the basis of 10-year CVD risk assessment using <10%, ≥10–<20%, and ≥20% as risk thresholds.

Abbreviations: CTCS, CT coronary artery calcium score.

Amongst the updated models, the FRS+CTCS had the highest NRI (Table 4). For the FRS updated with the other novel risk markers, the reclassification was limited and the NRI was close to zero for both CVD and CHD as end point (see Table 4 and Tables S4 and S5). Net reclassification improvement results were similar when using the <6, ≥6–<20%, ≥20% risk categorization. The number of high risk (≥20%) individuals reclassified to lower risk was limited –even for CTCS. Those who were reclassified upwards had a much higher 30-year CVD and CHD risk than the risk for those remaining in their risk category or who were reclassified downwards (Table 3 and Table S4a).

**Table 4:** Predictive value of novel risk markers for 10-year cardiovascular disease.

	FRS+CTCS	FRS+cIMT	FRS+CRP	FRS+ABI
**Δ C-statistic vs. FRS [95%CI]**	0.02 [0.01–0.03]	0.00 [0.00–0.01]	0.00 [0.00–0.01]	0.00 [0.00–0.00]
**NRI with <10%, ≥10–<20%, and ≥20%**				
NRI|event [ 95%CI ]	0.07 [−0.02–0.17]	0.00 [−0.02–0.03]	0.01 [−0.02–0.05]	−0.01 [−0.04–0.02]
NRI|no event [ 95%CI ]	0.02 [0.00–0.05]	0.01 [0.01–0.01]	0.00 [0.00–0.01]	0.01 [0.01–0.01]
NRI total [ 95%CI ]	0.10 [0.02–0.19]	0.01 [−0.01–0.04]	0.01 [−0.02–0.05]	0.00 [−0.03–0.03]
**NRI with <6%, ≥6–<20%, and ≥20%**				
NRI|event [ 95%CI ]	0.06 [−0.03–0.15]	−0.01 [−0.03–0.02]	0.00 [−0.03–0.04]	−0.01 [−0.03–0.01]
NRI|no event [ 95%CI ]	0.07 [0.05–0.09]	0.03 [0.03–0.03]	0.02 [0.01–0.02]	0.01 [0.01–0.01]
NRI total [ 95%CI ]	0.13 [0.05–0.22]	0.02 [−0.01–0.05]	0.02 [−0.01–0.06]	0.00 [−0.02–0.02]

Abbreviations: ABI, ankle-brachial index; cIMT, carotid intima-media thickness; CRP, high-sensitivity C-reactive protein; C-statistic, Harrell's concordance index; CTCS, CT coronary artery calcium score; FRS, Framingham risk score; NRI, net reclassification improvement.

Subjects who were traditionally classified as intermediate (≥10–<20%) 10-year CVD risk, were most frequently reclassified by CTCS. In this intermediate risk category, 0.39 (95%CI 0.23–0.55) of those with a CVD event within 10 years were reclassified upwards, whereas only 0.17 (95%CI 0.09–0.27) were reclassified downwards. For the subjects who did not experience an event, 0.37 (95%CI 0.35–0.39) were reclassified downwards and 0.18 (95%CI 0.11–0.25) upwards. The resulting bias-corrected NRI from updating FRS by CTCS in the intermediate risk category was 0.15 (95%CI 0.05–0.27). Defining ≥6–<20% as the intermediate risk category, the bias-corrected NRI was 0.13 (95%CI 0.06–0.21). The C-statistic of the FRS increased most by adding CTCS (Table 4 and Table S5). It increased from 0.82 (95%CI 0.79–0.85) to 0.84 (95%CI 0.81–0.86) for predicting CVD and from 0.84 (95%CI 0.82–0.86) to 0.87 (95%CI 0.84–0.89) for predicting CHD.

## Discussion

In this study, we modeled the predictive value of adding four novel cardiovascular risk markers to traditional Framingham risk scores (FRSs) in individuals representative of the U.S. general population. Whereas previous studies have focused on the predictive value of risk markers in specific longitudinal cohorts, we aimed to study the potential value of using risk markers in the US population as a whole. We used the two most commonly used endpoints 10-year CVD and CHD risk, together with two recommended risk categorization methods: <10%, 10–19%, ≥20% and <6%, 6–19%, ≥20% for low, intermediate, and high risk respectively. Among the four updated risk scores, the FRS updated with CTCS showed the most impact on reclassification for both CVD and CHD as endpoint, regardless of the risk thresholds used. Most reclassification occurred in those traditionally at intermediate risk; in other risk categories reclassification was less evident. FRS updated by cIMT, CRP and ABI had limited value with regard to appropriate reclassification and improvement of the C-statistic.

Previous cohort studies have demonstrated the added predictive value of CT coronary artery calcium score (CTCS), carotid intima-media thickness (cIMT), high-sensitivity C-reactive protein (CRP), and the ankle-brachial index (ABI) beyond FRS. The latter three risk markers were recently evaluated in large individual-level meta-analyses combining data from several cohort studies [12], [13], [23], [25]. Although the meta-analyses showed that these markers are associated with CVD independently from Framingham risk factors, the impact on improving risk prediction and classification was generally limited. The meta-analysis evaluating cIMT for 10-year CVD prediction showed similar C-statistics for the FRS: 0.757, and FRS with addition of common cIMT: 0.759. Only a small NRI: 0.008 was observed in the total population, which increased to 0.036 in individuals at intermediate risk [13]. This meta-analysis did not include recently published Framingham Study data that showed similar results: a small change in the C-statistic: 0.748 to 0.751 and 0.0 NRI. The meta-analysis on CRP showed a change in the C-statistic of 0.0039, and the NRI was 0.0152 for CVD prediction. The Framingham Offspring data included within the analysis showed that the C-statistic of 0.7779 increased by 0.0040. In the other included cohort studies, changes in the C-statistic varied from −0.0027 to 0.0157 [25]. In the meta-analysis on ABI, CHD risks were calculated after cross-tabulating a FRS for predicting 10-yr CHD risk categories by four different ABI categories. Meaningful reclassification by ABI was limited to women only: 7% of women at low risk and 10% of the women at intermediate risk were reclassified as high risk based on an ABI≤0.90 [23]. Changes in the C-statistic and NRI with ABI≤0.90 have not been established. A recent study in the Atherosclerosis Risk in Communities Study (ARIC Study) showed only modest improvement in the C-statistic: 0.756 to 0.758 and a NRI of 0.008 [26]. For CTCS, individual-level meta-analyses have not yet been conducted, although a systematic review of cohort studies shows that the impact on the C-statistic and NRI is generally larger: changes in the C-statistic varied from 0.04 to 0.13 and NRIs varied from 0.14 to 0.25 [9]. The four risk markers were evaluated in a direct comparison by only two cohort studies: the Multi-Ethnic Study of Atherosclerosis (MESA) and the Rotterdam Study [17], [27]. Both studies concluded that among the four markers, CTCS has the most added value in those at intermediate risk. In MESA, addition of CTCS, cIMT, CRP or ABI to a FRS plus race/ethnicity led to NRIs of 0.659, 0.102, 0.079, and 0.036 respectively. In the Rotterdam Study, these were 0.393, 0.046, 0.092, and 0.073. These NRIs were, however, not bias-corrected [21].

Generalizing results on reclassification from cohort studies to the general population is not straightforward. The impact of a novel risk marker on improving risk classification is determined by the strength of the association with the outcome, but also depends on the joined distribution of the marker and traditional risk factors in the population [10]. Because the distribution of risk factors in cohort studies is not comparable to the general population, we reproduced cardiovascular risk predictions by Framingham risk factors and novel risk markers within a recent NHANES sample while hypothesizing that these are generalizable. Although we were able to apply the summarized independent associations of novel risk markers with CVD to the NHANES sample, our study bears some important limitations. First, the NHANES did not include measurements of CTCS and cIMT. We therefore had to impute these measurements. We used correlations between Framingham risk factors and the other two novel risk markers as observed in the Rotterdam Study for the imputation process. Thus, the CTCS and cIMT values were distributed in the NHANES subjects conditionally on the assumption that the correlations in the Rotterdam Study are applicable to the NHANES population. Second, the NHANES data do not include CVD event rates and we therefore had to assume that the FRS [18] would be valid for the NHANES population in predicting event rates. However, it has been shown that Framingham-based predictions perform fairly well in most U.S. subpopulations [28]. Third, for the simulation of CVD event rates, we assumed that the associations of the four novel risk markers with CVD were independent of each other. Few studies published the change in hazard ratios of these novel risk markers after subsequently adding them to the FRS. Generally, the amount of confounding is limited [29]. Fourth, because our purpose was to evaluate the additional value of novel risk markers in the light of competing risk by non-CVD death, we chose a FRS that took into account the competing risk of non-CVD death for our simulation model. This FRS however uses total CVD as outcome and does not allow associations of traditional risk factors to be different for CHD and stroke events [3]. We therefore hypothesized that these effects would be similar. Although this seems to be a reasonable assumption for the most important cardiovascular risk factors -age and sex, this may be less true for other risk factors such as lipid levels [30]. However, CHD comprises the major part of total CVD. This implies that the associations of the traditional risk factors with CVD are closer to that of CHD than of stroke, and the results for reclassification of CHD will be relatively unaffected by this assumption. Finally, putting CHD and stroke and under the same term might be problematic when the goal is to individualize predictions while considering the difference in pathophysiology. For example, cIMT might well improve predictions of future stroke but not CHD. A separate assessment of stroke risk is generally however not advocated by most guideline groups, and we therefore did not evaluate a potential improvement of stroke prediction [31].

Instead of a priori focusing on individuals at intermediate risk [14], [27], we also included low and high-risk individuals. In theory, reclassifying high-risk individuals without events downwards could be beneficial as well. However, we demonstrated that CTCS has the largest value in refining decision-making in the intermediate risk category. Reclassification of subjects originally at low or high risk was much more limited. The size of the U.S. general population considered to be at intermediate risk largely depends on the chosen outcome and risk thresholds. Thus, the potential impact of additional testing with novel risk markers to decrease the total number of events will vary with this definition. Its impact will also depend on the indirect association of the novel risk marker with competing non-CVD death, e.g. through a strong correlation with age. There is, however, no indication that those reclassified to high risk suffer from a larger risk of competing death as demonstrated by a concordant increase in long-term, 30-year risk. Ultimately, costs and effects of recommended preventive treatment on quality-adjusted life expectancy should be considered for evaluating the impact of novel cardiovascular risk assessment strategies [32].

In conclusion, among four promising novel risk markers, only CTCS is expected to have significant incremental predictive value in the U.S. general population, and especially in those at intermediate risk. Future research should be performed to evaluate the clinical impact and cost-effectiveness of various novel cardiovascular risk assessment strategies.

## Supporting Information

Text S1 **Supporting Text.**
(DOC)

Figure S1 **Forrest plot of hazard ratios of one unit increase in the natural logarithm of (CTCS+1) for CHD.** Estimated heterogeneity variance: 0.0023 p = 0.146.
(JPG)

Figure S2 **Forrest plot of hazard ratios of one unit increase in the natural logarithm of (CTCS+1) for stroke.** Estimated heterogeneity variance: 0.0069 p = 0.107.
(JPG)

Figure S3 **Forrest plot of hazard ratios of an ABI≤0.9 vs >0.9 for CHD.** Estimated heterogeneity variance: 0.032 p = 0.09.
(JPG)

Figure S4 **Forrest plot of hazard ratios of an ABI≤0.9 vs >0.9 for stroke.** Estimated heterogeneity variance: 0 p = 0.503.
(JPG)

Figure S5 **Schematic representation of the microsimulation state-transition model.**
(JPG)

Figure S6 **Comparison Original Framingham CVD estimations vs. Model's predictions over a 30-yr time horizon.**
(JPG)

Table S1 **Model Input Parameters.**
(DOCX)

Table S2 **General characteristics of included studies.**
(DOCX)

Table S3 **Ten-year cardiovascular disease (CVD) risk reclassification by cIMT, CRP, and ABI.**
(DOCX)

Table S4 **Ten-year coronary heart disease (CHD) risk reclassification tables.**
(DOCX)

Table S5 **Predictive value of novel risk markers for 10 yr coronary heart disease.**
(DOCX)
