# Supplementary material for: Predictive Value of Updating Framingham Risk Scores with Novel Risk Markers in the U.S. General Population
Source: PLoS One. 2014 Feb 18;9(2):e88312. doi: 10.1371/journal.pone.0088312 (PMC3928195; doi:10.1371/journal.pone.0088312)
Supplement: Text S1 — Supporting Text. (DOC) [file pone.0088312.s001.doc]

**Text S1**

**Meta-analyses**

To standardize the reported units and categories into the desired units for CTCS: natural logarithm of (CTCS + 1) and the categorization of ABI: ≤0.9 vs > 0.9, we assumed a log-linear relation between the hazard of coronary heart disease (CHD) and stroke with the natural logarithm of (CTCS + 1) and continuous ABI up to a value of 1.4. If categories were reported, we performed linear regression on the log hazard ratios with the reported median values of each category as co-variables to derive relative risks on a continuous scale. If medians were not reported for each category we estimated them using the group mean and standard deviation assuming a normal distribution. Medians on the untransformed CTCS scale were taken assuming that the natural logarithm of the median would approximate the median of the natural logarithm of (CTCS + 1). Two studies reported the HR of log_2_(CTCS+1) instead of the natural logarithm, these were converted to the natural logarithm scale using a factor 1.4427.

**Imputation of CTCS and cIMT Values**

The Rotterdam Study is a population-based cohort study of individuals aged 55 years and older living in Rotterdam, the Netherlands . Demographics, traditional risk factors, CTCS, cIMT, hs-CRP, ABI, and information on cardioprotective drugs were measured during re-examination visits in a subset (n=1,915) of this cohort (for baseline characteristics see Table 1 in manuscript). Details on how these novel risk markers and the other variables were measured are published elsewhere .

First we imputed missing values of the traditional risk factors in the NHANES individuals (N=16,602), taking into account the according sample weights published by NHANES. Then we merged the imputed NHANES set with 1,915 individuals of the Rotterdam Study, including the novel risk markers. This extended set was bootstrapped with covariates age, sex, traditional risk factors, CVD history, cardioprotective drug information, and novel risk markers as input for the imputation algorithm. For imputation we have used the R ‘aregImpute’ function from the ‘Hmisc’ package. After the imputation procedure, we excluded NHANES subjects with prior CVD, NHANES subjects younger than 40 years of age and the Rotterdam study participants, leaving a study population of 3,736.

**Recalibration of Updated Framingham Risk Scores (FRS)**

We developed a state-transition model with three health states: Alive and CVD-free (Well), Post-CVD, and Dead (see Figure S5). One-year transition probabilities of Well 🡪 CVD and Well 🡪 Dead were based on the 30-year FRS, which calculates the cumulative incidence of CVD and competing non-CVD death. 30-year cumulative CVD incidence I_CVD_ is calculated by summing the product of CVD hazard h_CVD_ at failure time t_i_ and the survival of competing events S(t_i_-1) for all failure times up to 30 year follow-up:

We divided the baseline CVD-survival function into 2 survival functions: 1) coronary heart disease (CHD) and 2) stroke using the reported number of coronary heart disease and stroke events for men and women. The linear predictor of the 30-year FRS was extended with adjusted HRs of 4 novel risk markers based on systematic reviews of literature. Individual risk profiles including data on traditional and 4 novel risk factors were taken from 3,736 asymptomatic subjects of the National Health and Nutrition Examination Survey (NHANES) 2003-2004 examination round. To mimic survival selection of NHANES subjects at each time interval, we simulated cloned copies of NHANES subjects using Monte Carlo microsimulation within the state-transition model.

We followed a 4-step iterative calibration process:

1. The microsimulation model was run for cycle t, starting at the first year t=1, using the extended linear predictor values of NHANES subjects (uncalibrated simulated outcomes for cycle t)
2. The baseline CVD survival function was then recalibrated by a fixed term assuming that the average of the simulated outcomes during cycle t would equal the average calculated cumulative incidence based on the original FRS prediction (without the novel risk factors included) for cycle t.
3. The microsimulation model was then updated using the recalibrated CVD function for the next cycle t +1.
4. NHANES individuals who remained alive and CVD-free after the cycle t were selected for the recalibration step for the next period (transition from t=t to t=t+1).

For validation, we compared the cumulative CVD incidences of the microsimulation state-transition model at each year t with the cumulative CVD incidence calculated by the original FRS (Figure S6).

**Systematic Review Search Strategy**

**Inclusion criteria:**

**Population:** General (non-hospital) adult population free of hard coronary heart disease/ cardiovascular disease at baseline, not selected based by cardiovascular risk factors (e.g. renal disease, diabetes mellitus)

**Intervention:**Novel risk factor/biomarker + traditional "Framingham" risk factors: age, sex, total cholesterol, HDL cholesterol, systolic blood pressure, treatment for hypertension, smoking status, and diabetes mellitus

**Comparison:**Odds/risk/rate/hazard with and without biomarker adjusted for traditional

"Framingham" risk factors

**Outcomes:** 1) Hard coronary heart disease events: non-fatal myocardial infarction and fatal coronary heart disease

2) Non-fatal/fatal stroke

**Published:**1 September 2008 (ABI) / 1 July 2008 (CAC) – 19 April 2013

**Study type:**Cohort study or nested case-control study or case-cohort study or systematic review or meta-analysis of these study types

**Language:**English

**Pubmed Search Syntaxes**

**Coronary Artery Calcium**

1 cohort studies [MeSH Terms] OR cohort*[Text Word] OR controlled clinical trial [Publication Type]

2 case-control studies [MeSH Terms] OR (case*[Text Word]) AND control*[Text Word])

3 systematic [sb]

4 #1 OR #2 OR # 3

5 cardiovascular diseases [MeSH Terms]

6 coronary disease [MeSH Terms]

7 cardiovascular disease* [Title/Abstract]

8 coronary artery disease* [Title/Abstract]

9 coronary heart disease*[Title/Abstract]

10 #5 OR #6 OR #7 OR #8 OR #9

11 risk assessment [MeSH Terms]

12 risk factors [MeSH Terms]

13 prognosis [MeSH Terms]

14 risk factor* [Title/Abstract]

15 predict* [Title/Abstract]

16 Framingham [Title/Abstract] OR traditional [Title/Abstract] OR established [Title/Abstract] OR independent [Title/Abstract] OR conventional [Title/Abstract]

17 (#11 OR #12 OR #13 OR # 14 OR #15) AND #16

18 tomography, X-ray computed [MeSH Terms]

19 electron beam computed tomograph* [Text Word]

20 electron beam* [Text Word]

21 ebct [Text Word]

22 calcium scor* [Text Word]

23 coronary calcium [Text Word]

24 coronary artery calcium [Text Word]

25 cacs [Text Word]

26 #18 OR #19 OR #20 OR #21 OR #22 OR #23 OR #24 OR #25

27 #4 AND #10 AND #17 AND #26

28 #27 AND English[lang] AND ("2008/07/01"[PDAT] : "2015/01/01"[PDAT])

**Ankle Brachial Index**

1 cohort studies [MeSH Terms] OR cohort*[Text Word] OR controlled clinical trial [Publication Type]

2 case-control studies [MeSH Terms] OR (case*[Text Word]) AND control*[Text Word])

3 systematic [sb]

4 #1 OR #2 OR # 3

5 cardiovascular diseases [MeSH Terms]

6 coronary disease [MeSH Terms]

7 cardiovascular disease* [Title/Abstract]

8 coronary artery disease* [Title/Abstract]

9 coronary heart disease*[Title/Abstract]

10 #5 OR #6 OR #7 OR #8 OR #9

11 risk assessment [MeSH Terms]

12 risk factors [MeSH Terms]

13 prognosis [MeSH Terms]

14 risk factor* [Title/Abstract]

15 predict* [Title/Abstract]

16 Framingham [Title/Abstract] OR traditional [Title/Abstract] OR established [Title/Abstract] OR independent [Title/Abstract] OR conventional [Title/Abstract]

17 (#11 OR #12 OR #13 OR # 14 OR #15) AND #16

18 blood pressure [MeSH Terms] AND (ankle [Text Word] OR ankle [MeSH Terms])

19 ankle brachial blood pressure [Text Word]

20 ankle brachial pressure [Text Word]

21 ankle brachial index [Text Word]

22 abi [Text Word]

23 #18 OR #19 OR #20 OR #21 OR #22

24 #4 AND #10 AND #17 AND #23

25 #24 AND English[lang] AND ("2008/09/01"[PDAT] : "2015/01/01"[PDAT])

# References

# 1. Detrano R, Guerci AD, Carr JJ, Bild DE, Burke G, et al. (2008) Coronary calcium as a predictor of coronary events in four racial or ethnic groups. N Engl J Med 358: 1336-1345.

# 2. Mohlenkamp S, Lehmann N, Moebus S, Schmermund A, Dragano N, et al. (2011) Quantification of coronary atherosclerosis and inflammation to predict coronary events and all-cause mortality. J Am Coll Cardiol 57: 1455-1464.

# 3. Hofman A, van Duijn CM, Franco OH, Ikram MA, Janssen HL, et al. (2011) The Rotterdam Study: 2012 objectives and design update. Eur J Epidemiol 26: 657-686.

# 4. Elias-Smale SE, Proenca RV, Koller MT, Kavousi M, van Rooij FJ, et al. (2010) Coronary calcium score improves classification of coronary heart disease risk in the elderly: the Rotterdam study. J Am Coll Cardiol 56: 1407-1414.

# 5. Kavousi M, Elias-Smale S, Rutten JH, Leening MJ, Vliegenthart R, et al. (2012) Evaluation of newer risk markers for coronary heart disease risk classification: a cohort study. Ann Intern Med 156: 438-444.

# 6. Pencina MJ, D'Agostino RB, Sr., Larson MG, Massaro JM, Vasan RS (2009) Predicting the 30-year risk of cardiovascular disease: the framingham heart study. Circulation 119: 3078-3084.

# 7. Greenland P, LaBree L, Azen SP, Doherty TM, Detrano RC (2004) Coronary artery calcium score combined with Framingham score for risk prediction in asymptomatic individuals. Jama 291: 210-215.

# 8. Kondos GT, Hoff JA, Sevrukov A, Daviglus ML, Garside DB, et al. (2003) Electron-beam tomography coronary artery calcium and cardiac events: a 37-month follow-up of 5635 initially asymptomatic low- to intermediate-risk adults. Circulation 107: 2571-2576.

# 9. LaMonte MJ, FitzGerald SJ, Church TS, Barlow CE, Radford NB, et al. (2005) Coronary artery calcium score and coronary heart disease events in a large cohort of asymptomatic men and women. Am J Epidemiol 162: 421-429.

# 10. Wong ND, Gransar H, Shaw L, Polk D, Moon JH, et al. (2009) Thoracic aortic calcium versus coronary artery calcium for the prediction of coronary heart disease and cardiovascular disease events. JACC Cardiovasc Imaging 2: 319-326.

# 11. Elias-Smale SE, Wieberdink RG, Odink AE, Hofman A, Hunink MG, et al. (2011) Burden of atherosclerosis improves the prediction of coronary heart disease but not cerebrovascular events: the Rotterdam Study. Eur Heart J 32: 2050-2058.

# 12. Jain A, McClelland RL, Polak JF, Shea S, Burke GL, et al. (2011) Cardiovascular imaging for assessing cardiovascular risk in asymptomatic men versus women: the multi-ethnic study of atherosclerosis (MESA). Circ Cardiovasc Imaging 4: 8-15.

# 13. Abbott RD, Petrovitch H, Rodriguez BL, Yano K, Schatz IJ, et al. (2000) Ankle/brachial blood pressure in men >70 years of age and the risk of coronary heart disease. Am J Cardiol 86: 280-284.

# 14. Criqui MH, McClelland RL, McDermott MM, Allison MA, Blumenthal RS, et al. (2010) The ankle-brachial index and incident cardiovascular events in the MESA (Multi-Ethnic Study of Atherosclerosis). J Am Coll Cardiol 56: 1506-1512.

# 15. Lee AJ, Price JF, Russell MJ, Smith FB, van Wijk MC, et al. (2004) Improved prediction of fatal myocardial infarction using the ankle brachial index in addition to conventional risk factors: the Edinburgh Artery Study. Circulation 110: 3075-3080.

# 16. Newman AB, Shemanski L, Manolio TA, Cushman M, Mittelmark M, et al. (1999) Ankle-arm index as a predictor of cardiovascular disease and mortality in the Cardiovascular Health Study. The Cardiovascular Health Study Group. Arterioscler Thromb Vasc Biol 19: 538-545.

# 17. Abbott RD, Rodriguez BL, Petrovitch H, Yano K, Schatz IJ, et al. (2001) Ankle-brachial blood pressure in elderly men and the risk of stroke: the Honolulu Heart Program. J Clin Epidemiol 54: 973-978.

# 18. Hollander M, Hak AE, Koudstaal PJ, Bots ML, Grobbee DE, et al. (2003) Comparison between measures of atherosclerosis and risk of stroke: the Rotterdam Study. Stroke 34: 2367-2372.

# 19. Tsai AW, Folsom AR, Rosamond WD, Jones DW (2001) Ankle-brachial index and 7-year ischemic stroke incidence: the ARIC study. Stroke 32: 1721-1724.
