# Supplementary figures and images for: Predictive Value of Updating Framingham Risk Scores with Novel Risk Markers in the U.S. General Population
Source: PLoS One. 2014 Feb 18;9(2):e88312. doi: 10.1371/journal.pone.0088312 (PMC3928195; doi:10.1371/journal.pone.0088312)

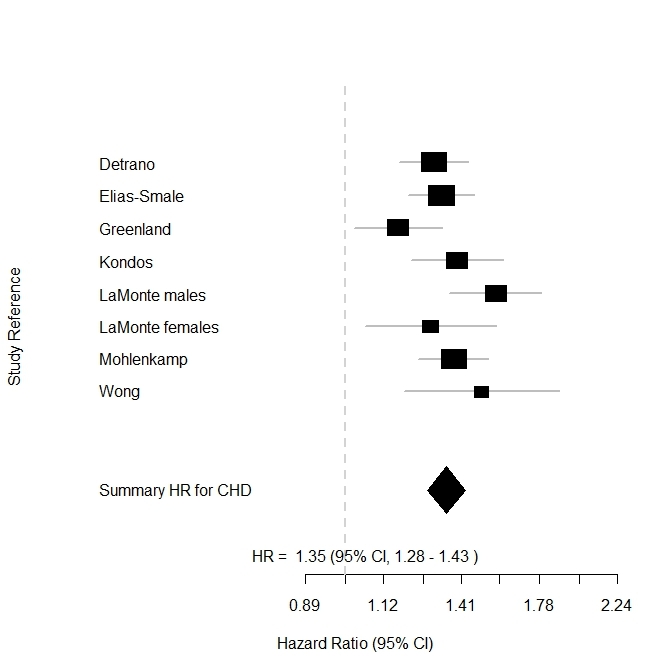

Supplement: Figure S1 — Forrest plot of hazard ratios of one unit increase in the natural logarithm of (CTCS+1) for CHD. Estimated heterogeneity variance: 0.0023 p = 0.146. (JPG) [file pone.0088312.s002.jpg]

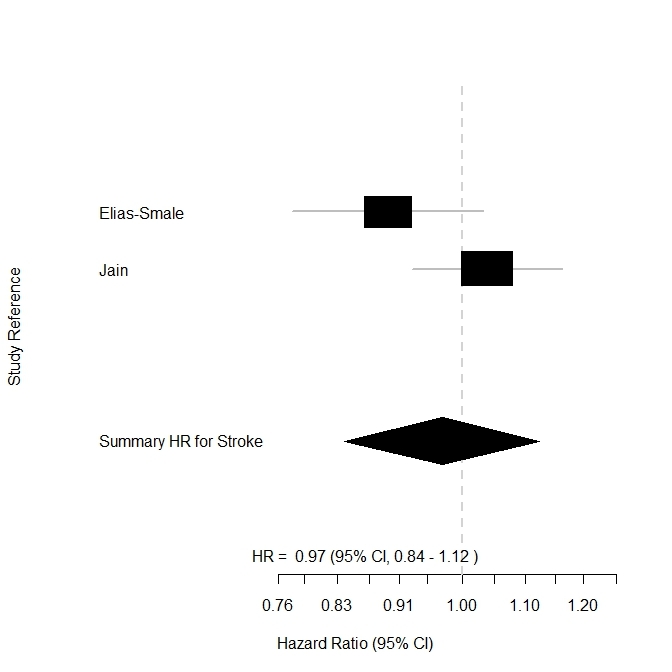

Supplement: Figure S2 — Forrest plot of hazard ratios of one unit increase in the natural logarithm of (CTCS+1) for stroke. Estimated heterogeneity variance: 0.0069 p = 0.107. (JPG) [file pone.0088312.s003.jpg]

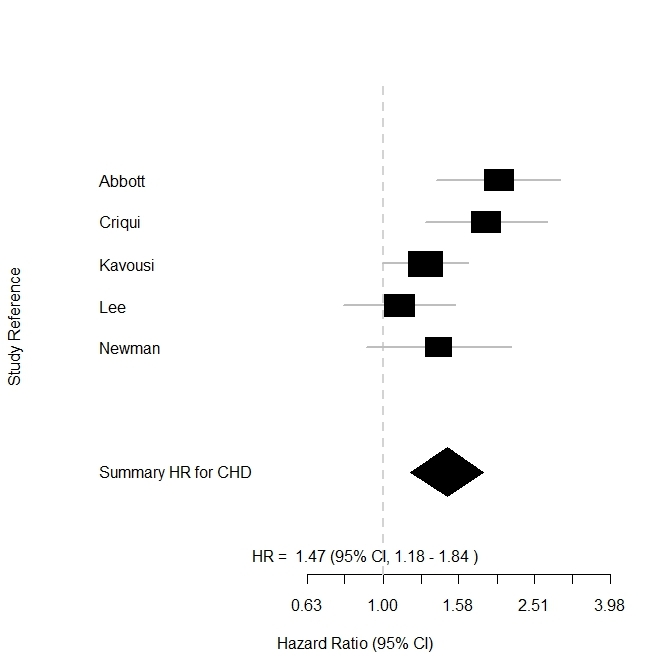

Supplement: Figure S3 — Forrest plot of hazard ratios of an ABI≤0.9 vs >0.9 for CHD. Estimated heterogeneity variance: 0.032 p = 0.09. (JPG) [file pone.0088312.s004.jpg]

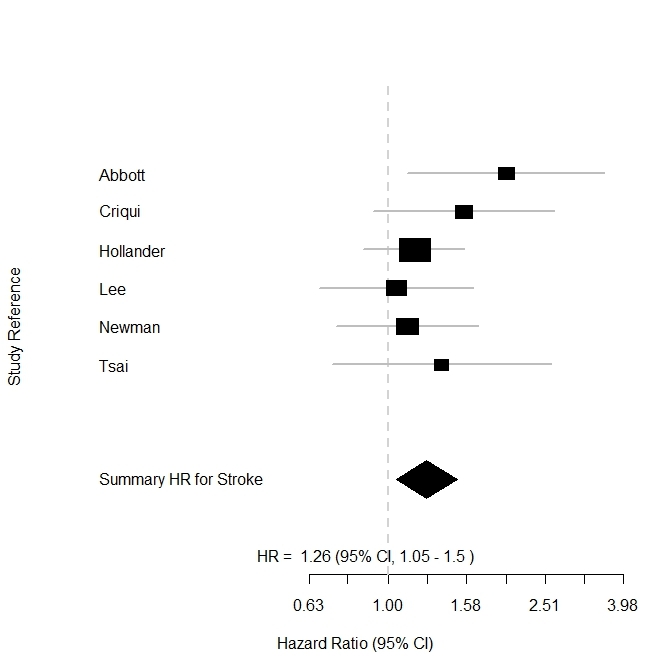

Supplement: Figure S4 — Forrest plot of hazard ratios of an ABI≤0.9 vs >0.9 for stroke. Estimated heterogeneity variance: 0 p = 0.503. (JPG) [file pone.0088312.s005.jpg]

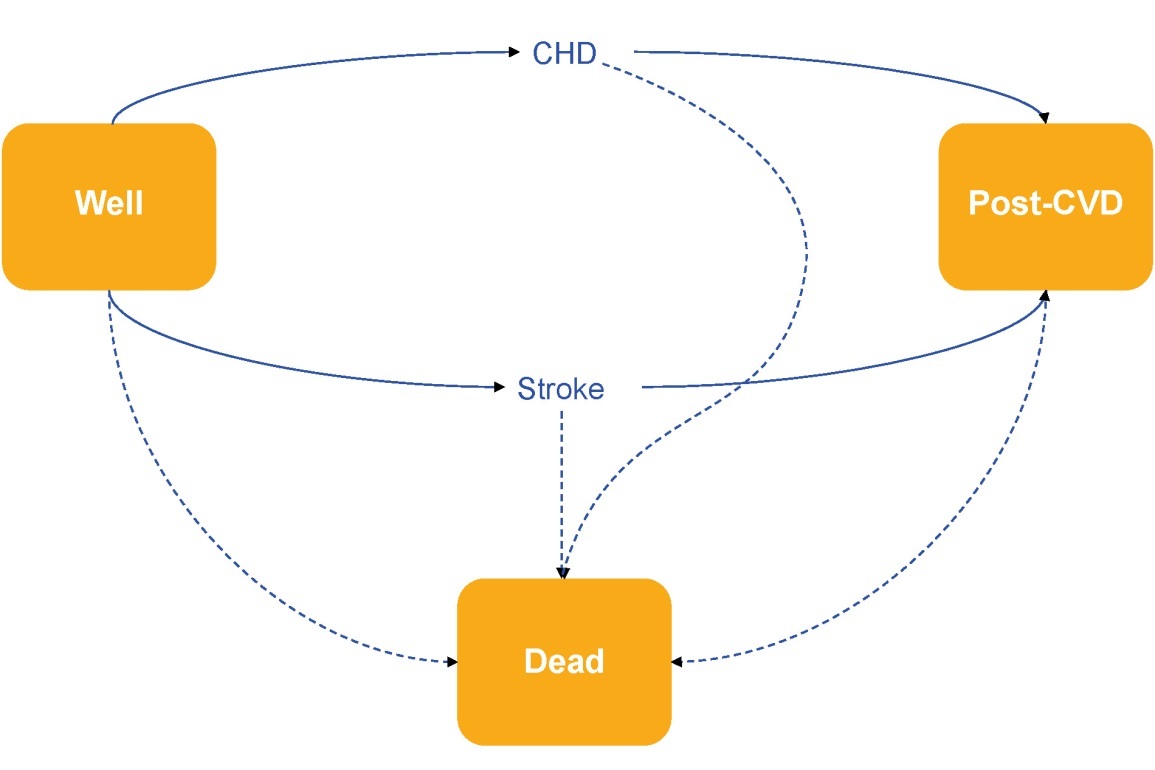

Supplement: Figure S5 — Schematic representation of the microsimulation state-transition model. (JPG) [file pone.0088312.s006.jpg]

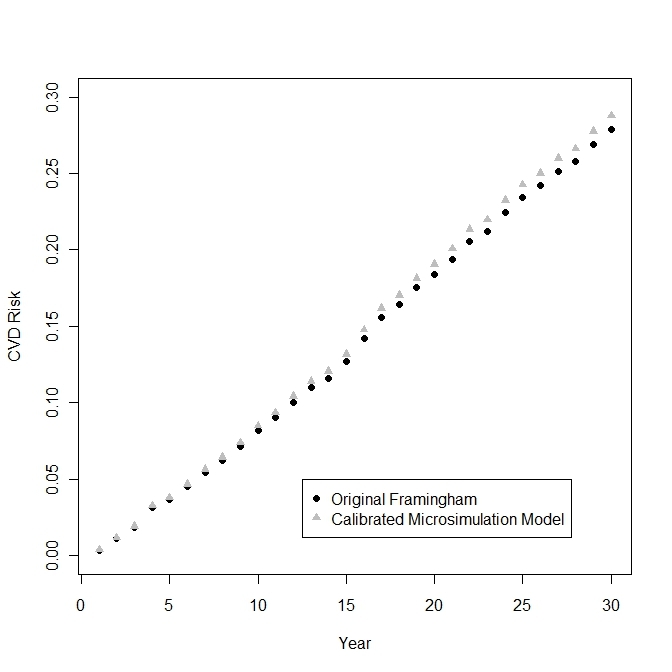

Supplement: Figure S6 — Comparison Original Framingham CVD estimations vs. Model's predictions over a 30-yr time horizon. (JPG) [file pone.0088312.s007.jpg]
