# Supplementary material for: Predictive Value of Updating Framingham Risk Scores with Novel Risk Markers in the U.S. General Population
Source: PLoS One. 2014 Feb 18;9(2):e88312. doi: 10.1371/journal.pone.0088312 (PMC3928195; doi:10.1371/journal.pone.0088312)
Supplement: Table S1 — Model Input Parameters. (DOCX) [file pone.0088312.s008.docx]

**Table S1. Model Input Parameters**

| **Parameter** | **Estimate** | **Source** |
| --- | --- | --- |
| One-year probability of having a first CHD event | Depending on cumulative CVD hazard function, and updated FRS | Pencina et al.[[6](#_ENREF_6)]  Pooled estimates for hazard ratios of the four novel risk markers from the systematic review |
| One-year probability of having a first stroke event | Depending on cumulative CVD hazard function, CHD: stroke event ratio 104/348 for men and 86/133 for women, and updated FRS | Pencina et al.[[6](#_ENREF_6)]  Pooled estimates for hazard ratios of the four novel risk markers from the systematic review |
| One-year probability of dying from non-cardiovascular mortality | Depending on cumulative hazard function for non-CVD death and original hazard ratios from the Framingham study | Pencina et al.[[6](#_ENREF_6)] |
