# Supplementary material for: Predictive Value of Updating Framingham Risk Scores with Novel Risk Markers in the U.S. General Population
Source: PLoS One. 2014 Feb 18;9(2):e88312. doi: 10.1371/journal.pone.0088312 (PMC3928195; doi:10.1371/journal.pone.0088312)
Supplement: Table S2 — General characteristics of included studies. (DOCX) [file pone.0088312.s009.docx]

**Table S2. General characteristics of included studies**

**a. CTCS – CHD studies**

| **First author, year** | **Country** | **Study population** | **N subjects** | **Mean age (years)** | **% Men** | **Follow-up duration** | **Predictor definition** | **N traditional risk factors used in multivariable analysis** | **Outcome** | **N events** |
| --- | --- | --- | --- | --- | --- | --- | --- | --- | --- | --- |
| Detrano, 2008[[1](#_ENREF_1)] | U.S. | MESA cohort | 6,722 | 62.2 | 47 | Median 3.9 years | Log_2_(CTCS+1) | 8 | Non-fatal MI and CHD death | 89 |
| Elias-Smale, 2010[[4](#_ENREF_4)] | The Netherlands | Rotterdam Study | 2,028 | 69.6 | 43 | Median 9.8 years | Ln(CTCS+1) | 8 | Non-fatal MI and CHD death | 135 |
| Greenland, 2004[[7](#_ENREF_7)] | U.S. | South Bay Heart Watch | 1,029 | 65.7 | 90 | Mean 6.3 years | Per 1-SD increase in CTCS (399) | 7 (summarized in ATPIII FRS) | Non-fatal MI and CHD death | 84 |
| Kondos, 2003[[8](#_ENREF_8)] | U.S. | Self-referred | 4,151 | 51 | 100 | Mean 3.1 years | CTCS per quartile | 5 | Non-fatal MI and CHD death | 52 |
| LaMonte, 2005[[9](#_ENREF_9)] | U.S. | Preventive health exam and self-referred | 10,746 total  6,835 men  3,911 women | 53.8 total  53.5 men  54.2 women | 64 | Mean 3.5 years | no detectable CTCS and sex-specific CTCS thirds | 5 | Non-fatal MI and CHD death | 81 total |
| Mohlenkamp, 2011[[2](#_ENREF_2)] | Germany | Heinz Nixdorf Recall (HNR) study | 3,966 | 59.3 | 47 | Median 5.0 years | Log_2_(CTCS+1) | 6 (included in FRS) | Non-fatal MI and CHD death | 91 |
| Wong, 2009[[10](#_ENREF_10)] | U.S. | Self-referred or referred by physician, enrolees of EISNER study | 2,303 | 56 | 62 | Mean 4.4 years | Ln(CTCS+1) | 7 (summarized in ATPIII FRS), if diabetic score of 20% or FRS if higher | Non-fatal MI and CHD death | 16 |

**b. CTCS – Stroke studies**

| **First author, year** | **Country** | **Study population** | **N subjects** | **Mean age (years)** | **% Men** | **Follow-up duration** | **Predictor definition** | **N traditional risk factors used in multivariable analysis** | **Outcome** | **N events** |
| --- | --- | --- | --- | --- | --- | --- | --- | --- | --- | --- |
| Elias-Smale, 2011[[11](#_ENREF_11)] | The Netherlands | Rotterdam Study | 2,153 | 69.2 | 45 | Median 3.5 years | CTCS per tertile | 8 | TIA and fatal or non-fatal ischemic stroke | 52 |
| Jain, 2011[[12](#_ENREF_12)] | U.S. | MESA | 4,965 | 61.5 | 48 | Median 5.8 years | Per 1-SD increase of Ln(CAC+1) | 8 | Fatal or non-fatal stroke | 65 |

**c. ABI – CHD studies**

| **First author, year** | **Country** | **Study population** | **N subjects** | **Mean age (years)** | **% Men** | **Follow-up duration** | **Predictor definition** | **N traditional risk factors used in multivariable analysis** | **Outcome** | **N events** |
| --- | --- | --- | --- | --- | --- | --- | --- | --- | --- | --- |
| Abbott, 2000[[13](#_ENREF_13)] | Hawaii | Honolulu Heart Program | 2,767 | 77.8* | 100 | 6 years follow-up | ABI < 0.8 and ABI 0.8 ≤ ABI < 1.0 vs. ABI ≥ 1.0 | 6 | Non-fatal MI and CHD death | 186 |
| Criqui, 2010[[14](#_ENREF_14)] | U.S. | Multi-Ethnic Study of Atherosclerosis | 6,647 | 62.0* | 47 | Median 4.8 years  Mean 5.3 years  Max 6.5 years | ABI < 1.0 vs. 1.0 ≤ ABI < 1.4 | 8 | Non-fatal MI, CHD  death, resuscitated cardiac arrest, and angina** | 226 |
| Kavousi, 2012[[5](#_ENREF_5)] | The Netherlands | Rotterdam Study | 5,933 | 69.1 | 41 | Median 6.8 years | ABI ≤0.9 vs 0.9 < ABI ≤ 1.4 | 8 | Non-fatal MI and CHD death | 347 |
| Lee, 2004[[15](#_ENREF_15)] | Scotland | Edinburgh Artery Study | 1,507 | Not reported (55-74) | Not reported | Not reported (more than 12 years) | ABI ≤0.9 vs 0.9 < ABI ≤ 1.5 | 7 | Non-fatal and fatal MI | 259 |
| Newman, 1999[[16](#_ENREF_16)] | U.S. | Cardiovascular Health Study | 4,268 | Not reported ( ≥ 65 years) | Not reported | Mean 5.1 years, 22 months black cohort  Max 6 years, 2 years black cohort | ABI < 0.9 vs. 0.9 ≤ ABI < 1.5 | 6 | Non-fatal and fatal MI | 188 |

**d. ABI – Stroke studies**

| **First author, year** | **Country** | **Study population** | **N subjects** | **Mean age (years)** | **% Men** | **Follow-up duration** | **Predictor definition** | **N traditional risk factors used in multivariable analysis** | **Outcome** | **N events** |
| --- | --- | --- | --- | --- | --- | --- | --- | --- | --- | --- |
| Abbott, 2001[[17](#_ENREF_17)] | Hawaii | Honolulu Heart Program | 2,767 | 77.8* | 100 | 6 years follow-up | ABI < 0.9 vs. 0.9 ≤ ABI ≤ 1.5 | 6 | Fatal or non-fatal stroke | 91 |
| Criqui, 2010[[14](#_ENREF_14)] | U.S. | Multi-Ethnic Study of Atherosclerosis | 6,647 | 62.0* | 47 | Median 4.8 years  Mean 5.3 years  Max 6.5 years | ABI < 1.0 vs. 1.0 ≤ ABI < 1.4 | 8 | Fatal or non-fatal stroke | 89 |
| Hollander, 2003[[18](#_ENREF_18)] | The Netherlands | Rotterdam Study | 6,913 | 69.5 | 39.7 | Mean 6.1 years | ABI ≤ 1.5 per tertile | 7 | Fatal or non-fatal stroke | 378 |
| Lee, 2004[[15](#_ENREF_15)] | Scotland | Edinburgh Artery Study | 1,507 | Not reported (55-74) | Not reported | Not reported (more than 12 years) | ABI ≤0.9 vs 0.9 < ABI ≤ 1.5 | 7 | Fatal or non-fatal stroke | 143 |
| Newman, 1999[[16](#_ENREF_16)] | U.S. | Cardiovascular Health Study | 4,268 | Not reported (≥ 65 years) | Not reported | Mean 5.1 years, 22 months black cohort  Max 6 years, 2 years black cohort | ABI < 0.9 vs. 0.9 ≤ ABI < 1.5 | 6 | Fatal or non-fatal stroke | 110 |
| Tsai, 2001[[19](#_ENREF_19)] | U.S. | Atherosclerosis  Risk In Communities Study | 14,839 | Not reported (≥ 45 years) | 45 | Median 7.2 years | ABI < 0.9 vs. ABI ≥ 0.9 | 7 | Fatal or non-fatal ischemic stroke | 206 |
