# Supplementary material for: Predictive Value of Updating Framingham Risk Scores with Novel Risk Markers in the U.S. General Population
Source: PLoS One. 2014 Feb 18;9(2):e88312. doi: 10.1371/journal.pone.0088312 (PMC3928195; doi:10.1371/journal.pone.0088312)
Supplement: Table S3 — Ten-year cardiovascular disease (CVD) risk reclassification by cIMT, CRP, and ABI. (DOCX) [file pone.0088312.s010.docx]

# Table S3. Ten-year cardiovascular disease (CVD) risk reclassification by cIMT, CRP, and ABI a. cIMT

|  | **FRS + cIMT** | | | **Overall** |
| --- | --- | --- | --- | --- |
| **FRS** | **<10%** | **≥10-<20%** | **≥20%** |  |
|  |  |  |  |  |
| **<10%** |  |  |  |  |
| N | 2604.21 | 36.79 | 0 | 2641 |
| % Events [95% CI] |  |  |  |  |
| 10 yr CVD | 2.8 [2.2 - 3.4] | 11.5 [2.4 - 21.4] | NA | 2.9 [2.3 - 3.5] |
| 30 yr CVD | 16 [14.5 - 17.6] | 46.4 [ 30.1 - 59.5 ] | NA | 16.4 [15.0 – 18.0] |
| **≥10- <20%** |  |  |  |  |
| N | 65.09 | 599.27 | 32.64 | 697 |
| % Events [95% CI] |  |  |  |  |
| 10 yr CVD | 7.5 [1.7 - 12.9] | 13.5 [11.1 - 16.2] | 21.9 [ 10.3 - 37.7 ] | 13.3 [ 10.8 - 16 ] |
| 30 yr CVD | 37.3 [28.2 - 50.1] | 48.8 [44.0 - 53.8] | 61.1 [ 44.6 - 77.2 ] | 48.3 [ 43.6 - 51.9 ] |
| **≥20%** |  |  |  |  |
| N | 0 | 30.77 | 367.23 | 398 |
| % Events [95% CI] |  |  |  |  |
| 10 yr CVD | NA | 17 [4.8 - 31.1] | 35.9 [ 30 - 42.6 ] | 34.4 [ 28.8 - 40.6 ] |
| 30 yr CVD | NA | 53.3 [38.1 – 69.0] | 69.5 [ 64.2 - 73.4 ] | 68.2 [ 63.2 - 72 ] |
|  |  |  |  |  |
| **Overall** |  |  |  |  |
| N | 2669.3 | 666.83 | 399.87 | 3736 |
| % Events [95% CI] |  |  |  |  |
| 10 yr CVD | 2.9 [2.4 - 3.5] | 13.5 [11.2 - 16.4] | 34.8 [29.1 - 41.4] | 8.2 [7.3 - 41.4] |
| 30 yr CVD | 16.5 [15.0 - 18.1] | 48.8 [44.5 - 52.9] | 68.8 [63.7 – 73.0] | 27.9 [26.3 – 73.0] |

**b. CRP**

|  | **FRS + CRP** | | | **Overall** |
| --- | --- | --- | --- | --- |
| **FRS** | **<10%** | **≥10-<20%** | **≥20%** |  |
|  |  |  |  |  |
| **<10%** |  |  |  |  |
| N | 2556.88 | 84.12 | 0 | 2641 |
| % Events [95% CI] |  |  |  |  |
| 10 yr CVD | 2.7 [2.0 - 3.3 ] | 9.6 [4.5 - 14.9] | NaN [NA - NA] | 2.9 [2.3 - 3.5] |
| 30 yr CVD | 15.5 [14.0 - 17.1] | 42.9 [33.0 - 51.6] | NaN [NA - NA] | 16.4 [15.0 – 18.0] |
| **≥10- <20%** |  |  |  |  |
| N | 101.76 | 546.83 | 48.41 | 697 |
| % Events [95% CI] |  |  |  |  |
| 10 yr CVD | 8.2 [3.3 - 13.1] | 13.4 [ 10.6 - 16.4] | 23.6 [10.9 - 34.6] | 13.3 [10.8 – 16.0] |
| 30 yr CVD | 36.7 [27.9 - 44.3] | 48.7 [44.4 - 52.8] | 68.6 [56.7 - 80.3] | 48.3 [ 43.6 - 51.9] |
| **≥20%** |  |  |  |  |
| N | 0 | 44.6 | 353.4 | 398 |
| % Events [95% CI] |  |  |  |  |
| 10 yr CVD | NaN [NA - NA] | 18.8 [ 8.9 – 33.0] | 36.4 [ 30.7 - 42.2] | 34.4 [ 28.8 - 40.6] |
| 30 yr CVD | NaN [NA - NA] | 53.8 [ 43.2 - 65.2] | 70 [ 64.6 - 73.9] | 68.2 [ 63.2 – 72.0] |
|  |  |  |  |  |
| **Overall** |  |  |  |  |
| N | 2658.64 | 675.55 | 401.81 | 3736 |
| % Events [95% CI] |  |  |  |  |
| 10 yr CVD | 2.9 [2.3 - 3.4] | 13.2 [10.7 – 16.0] | 34.9 [ 29.1 - 40.6] | 8.2 [ 7.3 - 40.6] |
| 30 yr CVD | 16.3 [14.7 - 17.9] | 48.3 [43.8 - 52.1] | 69.8 [ 64.5 – 74.0] | 27.9 [ 26.3 – 74.0] |

**c. ABI**

|  | **FRS + ABI** | | | **Overall** |
| --- | --- | --- | --- | --- |
| **FRS** | **<10%** | **≥10-<20%** | **≥20%** |  |
|  |  |  |  |  |
| **<10%** |  |  |  |  |
| N | 2616.7 | 24.3 | 0 | 2641 |
| % Events [95% CI] |  |  |  |  |
| 10 yr CVD | 2.8 [2.2 - 3.4] | 10.6 [0 - 23.1] | NA | 2.9 [2.3 - 3.5] |
| 30 yr CVD | 16.1 [14.7 - 17.7] | 47.6 [28.7 – 68.0] | NA | 16.4 [15.0 – 18.0] |
| **≥10- <20%** |  |  |  |  |
| N | 42.46 | 638.3 | 16.24 | 697 |
| % Events [95% CI] |  |  |  |  |
| 10 yr CVD | 8.8 [1.1 - 16.5] | 13.3 [10.6 - 16.2] | 23 [ 0 - 46.6 ] | 13.3 [10.8 – 16.0] |
| 30 yr CVD | 39.5 [25.3 - 53.5] | 48.4 [43.6 - 52.2] | 63.9 [41.4 - 85.7] | 48.3 [43.6 - 51.9] |
| **≥20%** |  |  |  |  |
| N | 0 | 30.44 | 367.56 | 398 |
| % Events [95% CI] |  |  |  |  |
| 10 yr CVD | NA | 20.3 [6.6 - 33.9] | 35.6 [29.8 - 41.7] | 34.4 [28.8 - 40.6] |
| 30 yr CVD | NA | 57.1 [42.6 – 73.0] | 69.1 [64.3 - 72.9] | 68.2 [ 63.2 – 72.0] |
|  |  |  |  |  |
| **Overall** |  |  |  |  |
| N | 2659.16 | 693.04 | 383.8 | 3736 |
| % Events [95% CI] |  |  |  |  |
| 10 yr CVD | 2.9 [2.4 - 3.5] | 13.6 [11.2 - 16.9] | 35.1 [29.3 - 41.4] | 8.2 [7.3 - 41.4] |
| 30 yr CVD | 16.5 [15.0 - 18.1] | 48.8 [44.2 - 52.8] | 68.9 [64.0 - 73.2] | 27.9 [26.3 - 73.2] |

Classification on the basis of 10 yr CVD risk - i.e. combined endpoint of CHD and Stroke – assessment using <10%, ≥10-<20%, and ≥20% as risk thresholds

Abbreviations: ABI, ankle-brachial index; cIMT, carotid intima-media thickness; CRP, high-sensitivity C-reactive protein; CVD = cardiovascular disease, FRS = Framingham risk score.
