# Supplementary material for: Predictive Value of Updating Framingham Risk Scores with Novel Risk Markers in the U.S. General Population
Source: PLoS One. 2014 Feb 18;9(2):e88312. doi: 10.1371/journal.pone.0088312 (PMC3928195; doi:10.1371/journal.pone.0088312)
Supplement: Table S4 — Ten-year coronary heart disease (CHD) risk reclassification tables. (DOCX) [file pone.0088312.s011.docx]

# Table S4. Ten-year coronary heart disease (CHD) risk reclassification tables

**a. CTCS**

|  | **FRS + CTCS** | | | **Overall** |
| --- | --- | --- | --- | --- |
| **FRS** | **<10%** | **≥10-<20%** | **≥20%** |  |
|  |  |  |  |  |
| **<10%** |  |  |  |  |
| N | 2828 | 158 | 13 | 2999 |
| % Events [ 95% CI ] |  |  |  |  |
| 10 yr CHD | 2 [1.4 - 2.3] | 12.8 [7.6 - 18.2] | 28.1 [0 - 58.7] | 2.6 [2.1 - 3.3] |
| 30 yr CHD | 11.5 [10.5 - 12.4] | 47.7 [38.8 - 55.6] | 70.2 [33.3 - 100] | 13.7 [12.3 – 15.0] |
| **≥10- <20%** |  |  |  |  |
| N | 191 | 180 | 154 | 525 |
| % Events [ 95% CI ] |  |  |  |  |
| 10 yr CHD | 5.6 [2.4 - 8.9] | 13.8 [8.8 - 19.3] | 27.7 [21 - 36.1] | 15.0 [11.5 - 19.1] |
| 30 yr CHD | 24.2 [18.8 - 29.8] | 47.1 [40.6 - 53.6] | 64.5 [55.4 - 70.8] | 43.9 [39.7 - 47.8] |
| **≥20%** |  |  |  |  |
| N | 16 | 37 | 159 | 212 |
| % Events [ 95% CI ] |  |  |  |  |
| 10 yr CHD | 7.7 [0 - 23.7] | 12.4 [2.6 – 24.0] | 42.4 [34.4 - 50.9] | 34.5 [27.4 - 41.3] |
| 30 yr CHD | 25.2 [6.2 – 50.0] | 36.9 [23.2 - 52.6] | 66.3 [56.9 - 74.2] | 58 [51.9 - 64.2] |
|  |  |  |  |  |
| **Overall** |  |  |  |  |
| N | 3035 | 376 | 325 | 3736 |
| % Events [ 95% CI ] |  |  |  |  |
| 10 yr CHD | 2.2 [1.6 - 2.7] | 13.2 [9.4 - 16.7] | 34.9 [29.0 - 40.7] | 6.2 [5.2 - 40.7] |
| 30 yr CHD | 12.4 [11.3 - 13.3] | 46.4 [41.1 - 50.8] | 65.5 [58.4 - 71.4] | 20.4 [19.1 - 71.4] |

**b. cIMT**

|  | **FRS + cIMT** | | | **Overall** |
| --- | --- | --- | --- | --- |
| **FRS** | **<10%** | **≥10-<20%** | **≥20%** |  |
|  |  |  |  |  |
| **<10%** |  |  |  |  |
| N | 2968 | 30 | 0 | 2999 |
| % Events [95% CI] |  |  |  |  |
| 10 yr CHD | 2.5 [2.0 - 3.2] | 13.8 [4.9 - 24.1] | NA | 2.6 [2.1 - 3.3] |
| 30 yr CHD | 13.4 [12 - 14.7] | 38.8 [22.8 - 52.3] | NA | 13.7 [12.3 - 15] |
| **≥10- <20%** |  |  |  |  |
| N | 55 | 444 | 25 | 525 |
| % Events [95% CI] |  |  |  |  |
| 10 yr CHD | 9.3 [3.6 - 15.2] | 14.9 [11.5 - 19.4] | 28.1 [13.9 - 48.3] | 15.0 [11.5 - 19.1] |
| 30 yr CHD | 33.7 [20.9 - 44.7] | 44.4 [40.1 - 48.5] | 56.9 [39.3 - 72.6] | 43.9 [39.7 - 47.8] |
| **≥20%** |  |  |  |  |
| N | 0 | 11 | 201 | 212 |
| % Events [95% CI] |  |  |  |  |
| 10 yr CHD | NA | 19.4 [0 - 45.5] | 35.3 [28.1 - 42.9] | 34.5 [27.4 - 41.3] |
| 30 yr CHD | NA | 51.7 [27.3 - 81.8] | 58.4 [52.2 - 64.5] | 58 [51.9 - 64.2] |
|  |  |  |  |  |
| **Overall** |  |  |  |  |
| N | 3024 | 485 | 227 | 3736 |
| % Events [95% CI] |  |  |  |  |
| 10 yr CHD | 2.7 [2.1 - 3.3] | 14.9 [11.5 - 19.4] | 34.5 [27.2 - 41.3] | 6.2 [5.2 - 41.3] |
| 30 yr CHD | 13.8 [12.4 - 15.1] | 44.2 [40.0 - 48.4] | 58.2 [51.8 - 64.3] | 20.4 [19.1 - 64.3] |

**c. CRP**

|  | **FRS + CRP** | | | **Overall** |
| --- | --- | --- | --- | --- |
| **FRS** | **<10%** | **≥10-<20%** | **≥20%** |  |
|  |  |  |  |  |
| **<10%** |  |  |  |  |
| N | 2938 | 61 | 0 | 2999 |
| % Events [95% CI] |  |  |  |  |
| 10 yr CHD | 2.5 [2.0 - 3.1 ] | 11.8 [4.5 - 19.3] | NA | 2.6 [2.1 - 3.3] |
| 30 yr CHD | 13.1 [11.8 - 14.5] | 39.5 [30.4 - 50.4] | NA | 13.7 [12.3 – 15.0] |
| **≥10- <20%** |  |  |  |  |
| N | 77 | 428 | 20 | 525 |
| % Events [95% CI] |  |  |  |  |
| 10 yr CHD | 8.4 [2.6 - 15.6] | 15.2 [11.1 - 19.2] | 36.1 [16.2 - 57.3] | 15.0 [11.5 - 19.1] |
| 30 yr CHD | 31.6 [23.1 - 40.9] | 45.1 [40.5 - 49.3] | 64.6 [44.3 - 81.2] | 43.9 [39.7 - 47.8] |
| **≥20%** |  |  |  |  |
| N | 0 | 25 | 187 | 212 |
| % Events [95% CI] |  |  |  |  |
| 10 yr CHD | NA | 23.4 [8.0 - 42.9 ] | 36.0 [27.9 - 42.5] | 34.5 [27.4 - 41.3] |
| 30 yr CHD | NA | 53.9 [36.0 - 70.1] | 58.6 [ 52 - 65.1] | 58.0 [51.9 - 64.2] |
|  |  |  |  |  |
| **Overall** |  |  |  |  |
| N | 3015 | 514 | 207 | 3736 |
| % Events [95% CI] |  |  |  |  |
| 10 yr CHD | 2.6 [2.0 - 3.3] | 15.2 [11.3 - 18.9] | 35.9 [28.5 – 42.0] | 6.2 [5.2 – 42.0] |
| 30 yr CHD | 13.6 [12.3 -15.0] | 44.9 [40.8 – 49.0] | 59.2 [52.5 - 65.6] | 20.4 [19.1 - 65.6] |

**d. ABI**

|  | **FRS + ABI** | | | **Overall** |
| --- | --- | --- | --- | --- |
| **FRS** | **<10%** | **≥10 - <20%** | **≥20%** |  |
|  |  |  |  |  |
| **<10%** |  |  |  |  |
| N | 2984 | 15 | 0 | 2999 |
| % Events [95% CI] |  |  |  |  |
| 10 yr CHD | 2.6 [2.0 - 3.2] | 14.4 [0 - 37.2] | NA | 2.6 [2.1 - 3.3] |
| 30 yr CHD | 13.5 [12.3 - 14.9] | 43.1 [18.1 - 68.6] | NA | 13.7 [12.3 – 15.0] |
| **≥10 - <20%** |  |  |  |  |
| N | 46 | 452 | 27 | 525 |
| % Events [95% CI] |  |  |  |  |
| 10 yr CHD | 8.4 [2.0 - 16.7] | 14.7 [11.4 - 18.7 ] | 29.7 [10.0 - 48.3] | 15.0 [11.5 - 19.1] |
| 30 yr CHD | 31.5 [18.4 - 45.2] | 44.3 [39.7 - 48.6] | 56.4 [37.9 - 70.8] | 43.9 [39.7 - 47.8] |
| **≥20%** |  |  |  |  |
| N | 0 | 17 | 195 | 212 |
| % Events [95% CI] |  |  |  |  |
| 10 yr CHD | NA | 19.8 [5.4 - 38.2] | 35.8 [28.7 - 42.8] | 34.5 [27.4 - 41.3] |
| 30 yr CHD | NA | 55.7 [33.3 - 77.8] | 58.3 [52.3 - 64.2] | 58.0 [51.9 - 64.2] |
|  |  |  |  |  |
| **Overall** |  |  |  |  |
| N | 3030 | 484 | 222 | 3736 |
| % Events [95% CI] |  |  |  |  |
| 10 yr CHD | 2.7 [2.1 - 3.4] | 14.9 [11.5 - 18.7] | 35.1 [28.1 - 41.9] | 6.2 [5.2 - 41.9] |
| 30 yr CHD | 13.8 [12.5 - 15.2] | 44.7 [39.9 – 49.0] | 58.1 [51.6 - 63.7] | 20.4 [19.1 - 63.7] |

Classification on the basis of risk assessment using <10%, ≥10-<20%, and ≥20% as risk thresholds

Abbreviations: ABI, ankle-brachial index; cIMT, carotid intima-media thickness; CRP, high-sensitivity C-reactive protein; CTCS, CT coronary artery calcium score; CHD, coronary heart disease; FRS, Framingham risk score.
