# Supplementary material for: Predictive Value of Updating Framingham Risk Scores with Novel Risk Markers in the U.S. General Population
Source: PLoS One. 2014 Feb 18;9(2):e88312. doi: 10.1371/journal.pone.0088312 (PMC3928195; doi:10.1371/journal.pone.0088312)
Supplement: Table S5 — Predictive value of novel risk markers for 10 yr coronary heart disease. (DOCX) [file pone.0088312.s012.docx]

**Table S5. Predictive value of novel risk markers for 10 yr coronary heart disease**

|  | **FRS + CTCS** | **FRS + cIMT** | **FRS + CRP** | **FRS + ABI** |
| --- | --- | --- | --- | --- |
| **∆ C-statistic vs. FRS**  **[ 95%CI ]** | 0.03 [0.02 – 0.04] | 0.00 [0.00 – 0.01] | 0.00 [0.00 – 0.01] | 0.00 [0.00 – 0.00] |
|  |  |  |  |  |
| **NRI with <10%, ≥10-<20%, and ≥20%** |  |  |  |  |
| NRI│event [ 95%CI ] | 0.21 [0.08 – 0.32] | 0.02 [-0.01 – 0.05] | 0.01 [-0.03 – 0.05] | 0.01 [-0.02 – 0.05] |
| NRI│no event [ 95%CI ] | -0.01 [-0.03 – 0.015] | 0.00 [0.00 – 0.01] | 0.01 [0.00 – 0.01] | 0.01 [0.00 – 0.01] |
| NRI total [ 95%CI ] | 0.21 [0.09 – 0.30] | 0.02 [-0.01 – 0.05] | 0.02 [-0.02 – 0.06] | 0.02 [-0.01 – 0.05] |
|  |  |  |  |  |
| **NRI with <6%, ≥6 -<20%, and ≥20%** |  |  |  |  |
| NRI│event [ 95%CI ] | 0.16 [0.04 – 0.27] | 0.02 [0.01 – 0.05] | 0.01 [-0.03 – 0.04] | 0.01 [-0.02 – 0.05] |
| NRI│no event [ 95%CI ] | 0.05 [0.02 – 0.07] | 0.01 [0.01 – 0.01] | 0.01 [0.00 – 0.01] | 0.01 [0.01 – 0.01] |
| NRI total [ 95%CI ] | 0.21 [0.11 – 0.30] | 0.03 [0.00 – 0.06] | 0.01 [-0.02 – 0.05] | 0.02 [-0.01 – 0.06] |

ABI = ankle-brachial index, cIMT = carotid intima-media thickness, CRP= C-reactive protein, C-statistic = Harrell’s concordance index, CTCS = CT coronary artery calcium score, FRS = Framingham risk score, NRI = net reclassification improvement.
